# The relationship between oxidative stress and preeclampsia. The serum ischemia-modified albumin levels and thiol/disulfide homeostasis

**DOI:** 10.4274/tjod.galenos.2020.23682

**Published:** 2020-07-29

**Authors:** Taylan Onat, Demet Aydoğan Kırmızı, Emre Başer, Müjgan Ercan, Melike Demir Çaltekin, Serenat Yalçın, Mustafa Kara, Deniz Esinler, Ethem Serdar Yalvaç

**Affiliations:** 1 Bozok University Faculty of Medicine, Department of Obstetrics and Gynecology, Yozgat, Turkey; 2 Harran University Faculty of Medicine, Department of Medical Biochemistry, Şanlıurfa, Turkey; 3 University of Health Sciences Turkey Antalya Training and Research Hospital, Antalya, Turkey; 4 Ahi Evran University Faculty of Medicine, Department of Obstetric and Gynecology, Kırşehir, Turkey; 5 Medical Park Hospital, Ankara, Turkey

**Keywords:** Preeclampsia, hypertension, oxidative stress, thiol/disulfide, ischemia-modified albumin

## Abstract

**Objective:**

Preeclampsia (PE) is a dangerous complication of pregnancy and still a major cause of maternal-fetal morbidity and mortality. Its etiology remains largely unknown, but researchers have suggested oxidative stress-mediated inflammation for the same. The purpose of this study is to investigate the relationship between oxidative stress and PE as well as the usability of oxidative stress indicators such as serum ischemia-modified albumin (IMA) levels and thiol/disulfide balance in the prediction of PE.

**Materials and Methods:**

The study included 47 pregnant women with PE and 57 healthy pregnant women. We measured their serum IMA, native thiol, total thiol, and disulfide levels. Additionally, we determined the optimal cutoff values via the receiver operating characteristic curve analysis.

**Results:**

There were no differences between the two groups with respect to the maternal age, body mass index, gravida, and parity. The native and total thiol levels were found to be low when the disulfide and IMA levels were high in the patients with PE (p<0.05). When the IMA level was corrected by the albumin level (IMAR), the significant difference between the two groups disappeared. We also found that the native and total thiol concentrations were correlated with the systolic and diastolic blood pressures. The optimal cut-off values calculated for the prediction of PE were as follows: 178.45 µmol/L (with sensitivity of 72% and specificity of 83%) for native thiol, 232.55 µ mol/L (with a sensitivity of 75% and specificity of 85%) for total thiol, and 29.05 µmol/L (with sensitivity of 65% and specificity of 72%) for disulfide.

**Conclusion:**

The balance of thiol/disulfide may play a role in the pathogenesis of PE and could be used as a biological marker for PE.

**PRECIS:** The balance of thiol/disulfide may play a role in the pathogenesis of preeclampsia.

## Introduction

Preeclampsia (PE) is a leading cause of both maternal-fetal morbidity and mortality, and it affects about 5%-10% of pregnancies^(1)^. PE is characterized by gestational hypertension with one or more of the following conditions detected after ≥20 weeks’ gestation: proteinuria, acute kidney injury, liver dysfunction, hematologic features, neurologic features, and placental dysfunction^(2)^. Unfortunately, the only treatment for PE is still delivery, and PE causes iatrogenic preterm birth in about 15% of pregnancies^(3)^.

The etiology of PE is still unknown. It is believed that the defect starts from the implantation of the placenta. The oxidative damage that occurs in the placenta causes inflammation, apoptosis, and the release of anti-angiogenic agents and cytokines into maternal circulation. These placenta-originating substances that pass into the maternal circulation cause endothelial dysfunction, which is the main component of PE pathophysiology^(4)^. Additionally, it has been shown that physiological oxidative stress, which is present in the early stages of pregnancy, stimulates the normal cell differentiation and cytotrophoblast proliferation before the initiation of feto-maternal circulation; however, its role in the development of PE is still unknown^(5)^.

Albumin is a vital part of the antioxidant systems. Its structure undergoes changes during oxidative stress, which lead to a reduction in the metal binding capacity. Subsequently, it is transformed into a new molecule called ischemia-modified albumin (IMA)^(6)^. The formation of reactive oxygen species (ROS) and free radicals temporarily changes the N-terminal region of albumin, which causes an increase in IMA concentration^(7)^. Furthermore, it has been shown to increase in the healthy pregnant women and other pregnancy-related situations^(8,9,10)^.

Another antioxidant system is the thiol group consisting of sulfur and hydrogen attached to the carbon atoms^(11)^. The circulating thiol comprises albumin thiols, protein thiols, and thiols having a low molecular weight. Thiol groups are reversibly converted to the disulfide bonds in the presence of oxygen radicals. Thiol/disulfide homeostasis (TDH) is protected by the conversion between the thiol and disulfide groups^(12)^. TDH plays an important role in the mechanisms such as programmed cell death and antioxidant protection^(13,14)^.

In the literature, there are studies evaluating the levels of TDH and IMA in pregnancy complications such as gestational diabetes, hyperemesis gravidarum, PE, and intrauterine growth retardation^(9,15,16,17)^. Because PE remains an important obstetric complication, there have been ongoing studies on the biochemical markers for the early detection of this disease. The investigation of serum markers related to oxidative stress involved in the etiology of PE can be useful in this matter. Based on this observation, the purpose of this study is to investigate two antioxidants, serum IMA and TDH, and compare these markers between the patients with PE and healthy pregnant women in the control group.

## Materials and Methods

We conducted this study between May 2017 and September 2018 after receiving the approval of the local Ethics Committee at the Yozgat Bozok University Faculty of Medicine, Department of Obstetrics and Gynecology (April 18, 2017, 2017-11/01). Importantly, we obtained written informed consent from all the pregnant women who agreed to participate in the study.

The study group consisted of 47 patients who were diagnosed with PE according to the recommendations of the International Society for the Study of Hypertension in Pregnancy^(2)^. The control group consisted of 57 normotensive pregnant women (blood pressure less than 140/90 mmHg). The blood pressure of participants in the outpatient clinic was measured with an adult-type blood pressure monitor (Perfect Aneroid 48, ERKA, Germany), whereas the inpatient follow-up was performed by using a patient monitor (Vista 120, Dräger, Germany). A 5 mL of fasting venous blood sample was taken from all the participants for the analysis of biochemical parameters. In the study group, proteinuria was measured in a 24-hour urine protein test. We did not included pregnant women with a history of PE, chronic gestational hypertension, renal, hepatic and thyroid diseases, and type I and type II diabetes mellitus in this study.

TDH parameters were measured as described previously by Erel and Neselioglu^(18)^. The total and native thiol levels were measured by a fully automated spectrophotometric method by using an autoanalyzer-cobas^®^ 6000 analyzer series (Roche Diagnostic Corp., Mannheim, Germany). The amount of dynamic disulfide was calculated as half of the difference between the total thiol and native thiol levels. After the determination of native and total thiol levels, the disulfide amounts, disulfide/total thiol percentage ratios, native thiol/total thiol percentage ratios, and disulfide/native thiol percentage ratios were calculated.

The serum IMA level was evaluated by using the method proposed by Bar-Or et al.^(7)^. A 200-mL patient sample was taken and 50 mL of cobalt chloride (CoCl_2_^.^6H_2_O, 1 g/L) was added to it. This procedure was followed by vigorous mixing. The mixture was incubated for 10 minutes to ensure the binding of cobalt albumin. A 50-mL (1.5 mg/mL) amount of dithiothreitol was added as a coloring agent and later mixed. After a two-minute incubation period, 1.0 mL of sodium chloride (0.9%) was added. The absorbance of assay mixtures was read at a wavelength of 470 nm by using a spectrophotometer (Thermo Scientific, Madison, WI). A blank was similarly prepared with the exclusion of the dithiothreitol. The results were reported as absorbance units (ABSU). The serum IMA and IMA/albumin ratio (IMAR) was also calculated. Serum IMAR was expressed as absolute units per gram (ABSU/g) of albumin.

### Statistical Analysis

We performed statistical analysis by using the Statistics Package for Social Sciences software (ver. 20.0; SPSS Inc., Chicago, IL). Descriptive analyses were presented as mean ± standard deviation. We analyzed the fit of the variables to the normal distribution by visual (histogram) and analytical (Shapiro-Wilk test) methods. Importantly, we compared the data with normal distribution by using the Student’s t-test, whereas we compared the data without normal distribution by using the Mann-Whitney U test. The correlation coefficient was calculated by the Pearson or Spearman tests according to the normal distribution of data. We used the receiver operating characteristic (ROC) curve analysis to determine the optimal cutoff value. A p-value of less than 0.05 was considered significant within a 95% confidence interval.

## Results

We divided 104 pregnant women who agreed to participate in this study into two groups. Group 1, the study group, consisted of 47 pregnant women with PE, whereas group 2, the control group, consisted of 57 healthy pregnant women. Table 1 shows the descriptive data from the study and control groups. There was no difference with respect to maternal age, BMI, gravida, and parity between the groups (p>0.05). We compared systolic and diastolic blood pressures, aspartate aminotransferase, alanine aminotransferase, lactate dehydrogenase, creatinine levels and platelet counts, and determined a significant difference (p<0.05) between the groups as we expected (Table 1).

IMA, native thiol, total thiol, the disulfide and disulfide/native thiol ratio, the disulfide/total thiol ratio, and the native/total thiol ratio were found to be significantly different between the groups (p<0.05) (Table 1). The native and total thiol levels were found to be low when the disulfide and IMA levels were high in the patients with PE. When the IMA level was corrected by the albumin level (IMAR), the significant difference between the two groups disappeared. Further analysis was conducted within the PE group to determine whether the biochemical parameters showed any correlations with each other and with blood pressure (Table 2). Furthermore, a significant negative correlation between total thiol and systolic blood pressure [Spearman’s Rho Coefficient (rs) -0.519, p<0.001) and diastolic blood pressure (r_s_: -0.512, p<0.001)] was found. Additionally, there was a significant and moderate negative correlation between the native thiol and systolic blood pressure [r_s_: -0.495, p<0.001] and diastolic blood pressure (r_s_: -0.496, p<0.001).

**Figure 1:**
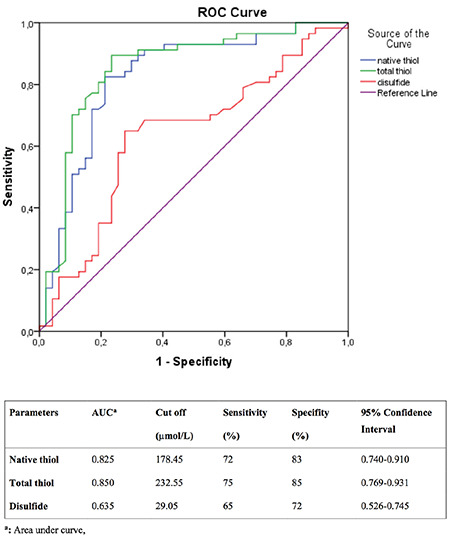
Receiver operating characteristic analysis (Specificity, Sensitivity, and the Cutoff Levels of Thiol/Disulfide Homeostasis in Preeclampsia)

We performed ROC analysis to evaluate the diagnostic performance of significant parameters in the prediction of PE (Figure 1). The optimal cutoff values were: native thiol, 178.45  µmol/L (with sensitivity of 72% and specificity of 83%); total thiol, 232.55  µmol/L (with 75% sensitivity and 85% specificity) and disulfide, 29.05  µmol/L (with sensitivity of 65% and specificity of 72%).

## Discussion

This case-control study investigated the relationship between PE and serum oxidative stress markers. It also found that the levels of native thiol, total thiol, and disulfide were significantly different in the PE group. Additionally, the levels of serum IMA were higher in the PE group as compared to the control group; however, this difference was eliminated after the correction for total albumin (IMAR) concentrations.

Despite decades of research, the pathogenesis and pathophysiology of PE are yet to be fully understood. Moreover, insufficient placentation at the beginning of pregnancy is believed to be the trigger for PE. The early 1990s witnessed the proposal of “two-stage model of PE” hypothesis^(19)^. In this hypothesis, PE consists of Stage 1 (pre-clinical stage) with inadequate placentation and Stage 2 (clinical stage) with maternal syndrome. It was not fully understood how PE progressed from stage 1 to stage 2. It was believed that endothelial dysfunction, which leads to the emergence of clinical picture, may be the link between stage 1 and 2^(20)^. While the two-stage model of PE hypothesis describes PE as a homogeneous disease, subsequent studies have shown that PE is a heterogeneous disease and that this hypothesis cannot explain some of the PE cases^(19,21)^. In early pregnancy, spiral arteries lose the muscle layer and adapt to pregnancy. The problems in the remodeling process affect the flow rate rather than the flow volume of the blood^(22)^. It has been shown that ischemia-reperfusion injury and oxidative stress caused by this high-pressure abnormal flow lead to more damage in the placenta than chronic hypoxia^(22)^. It has been suggested that severe oxidative stress is associated with maternal clinical symptoms of PE^(23)^.

Moreover, inadequate placentation causes the occurrence of ischemia-reperfusion injury and vasoactive agents such as soluble fms-like tyrosine kinase-1 and soluble endoglin^(24)^. These anti-angiogenic vasoactive agents show their effects through oxidative stress, induced systemic inflammation, and endothelial cell damage^(25,26)^. PE is characterized by a deterioration in the oxidant-antioxidant balance in the favor of oxidative stress^(27)^. ROS are the most important components of oxidative damage^(28)^. ROS shows their vasoconstrictive effects by inhibiting the endothelial-dependent vasodilation pathways (such as nitric oxide). This action suggests that oxidative stress play a pivotal role in the development of PE.

Thiol, also known as mercaptan, is an organic compound that contains the sulfhydryl groups^(11)^. The thiol/disulfide system, which is one of the most important antioxidant systems of the human body, can be oxidized in the presence of free oxygen radicals due to the thiol groups and can be reversibly converted to the sulfhydryl bonds^(29)^. TDH has been investigated for many physiological conditions such as apoptosis, antioxidant defense mechanisms as well as for pathological processes such as cancer, diabetes mellitus, intrauterine growth restriction, obstructive sleep apnea, and so on^(13,14,30,31,32,33)^. Hydrogen sulfide (H_2_S), a functional group of native thiol, regulates the vascular tone through nitric oxide^(34)^. As indicated by Holwerda et al.^(35)^, the production of H_2_S is less in the placental samples from the patients with PE. The oxidative damage from ROS on the vascular structures increases with the weakening of TDH. In PE, Ozler et al.^(15)^ first identified TDH and showed that TDH may play a role in the pathogenesis of PE. Native and total thiol levels were found to be low and the disulfide levels were high in patients with PE; these observations were similar to our results. Recent studies found a correlation between the severity of PE and the impairment of TDH^(36)^. As in line with the literature, our study showed that TDH significantly deteriorated in the favor of PE. Additionally, it also found that the native and total thiol concentrations were correlated with the systolic and diastolic blood pressures.

Although IMA levels were first studied in myocardial ischemia, they have been studied for many conditions, such as multiple sclerosis, PE, and acute appendicitis^(9,37,38)^. The IMA levels have showed contradictory results in previous studies conducted on PE. The study by Van Rijn et al.^(39)^ examined the IMA levels in three groups: 12 pregnant women with PE, 12 normotensive pregnant women, and 12 healthy non-pregnant women showed no significant difference. The study by Üstün et al.^(9) ^included 54 pregnant women and found that the IMA level was significantly higher in the PE group. Additionally, the severity of the disease and IMA levels were positively correlated in the same study. Studies investigating the level of IMA in healthy pregnant women and healthy non-pregnant women have shown that pregnancy is a factor that significantly increases the IMA level on its own^(8,39)^. In this study, the IMA level was significantly different in both the groups. However, this difference disappeared when the IMA level was corrected with albumin. The IMA levels during pregnancy are higher in pregnant women than in healthy non-pregnant women. A hypoxic environment is required to increase placentation. In our opinion, the hypoxic environment in the early period of pregnancy activates the cytotrophoblasts and increases the level of IMA.

### Study Limitations

This study has several limitations. First, the obstetric and neonatal outcomes could not be included due to the insufficiencies of neonatal intensive care units’ recording system. Second, most of these patients could not give birth in our center. Other limitations were the differences in the severity of PE among the study group and a small sample size. However, the evaluation of the parameters of two different antioxidant defense systems at the same time is a factor that adds weight to our research.

## Conclusion

Oxidative stress is an important part of the pathophysiology of PE. This study evaluated two different oxidative stress markers, and found that TDH was significantly different in the PE group, but there was no significant difference for IMAR. According to our hypothesis, TDH may play a role in the pathogenesis of PE and serum levels or ratios can be used to predict PE. To confirm these results, there is a need for future prospective randomized controlled studies researching the serum levels also in the early gestational weeks with higher numbers of subjects and groups.

## Figures and Tables

**Table 1:**
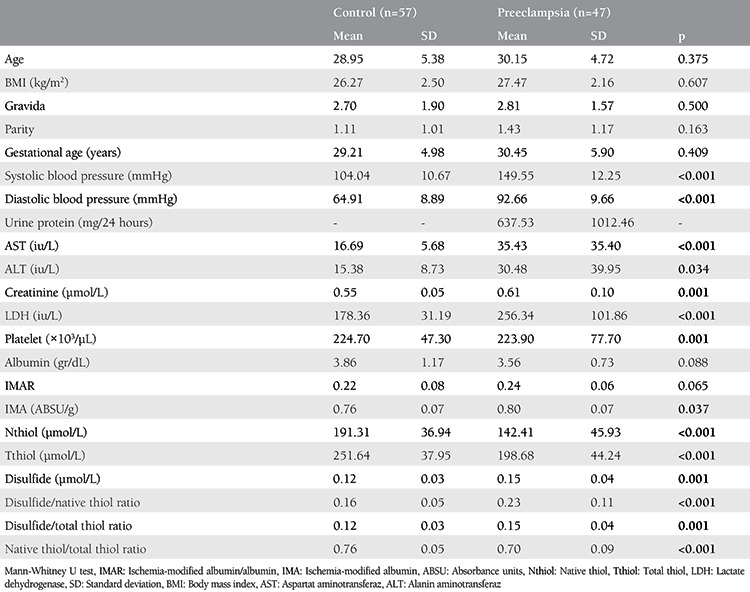
The Characteristics of the preeclampsia and control groups

**Table 2:**
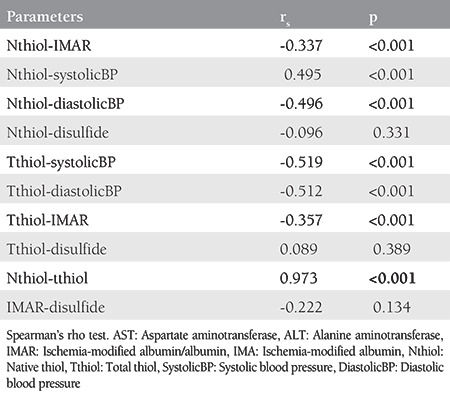
Correlations of biochemical parameters

## References

[1] North RA, McCowan LM, Dekker GA, Poston L, Chan EH, Stewart AW, et al (2011). Clinical risk prediction for pre-eclampsia in nulliparous women: Development of model in international prospective cohort. BMJ.

[2] Brown MA, Magee LA, Kenny LC, Karumanchi SA, McCarthy FP, Saito S, et al (2018). Hypertensive Disorders of Pregnancy: ISSHP Classification, Diagnosis, and Management Recommendations for International Practice. Hypertension.

[3] Joern H, Rath W (2000). Correlation of Doppler velocimetry findings in twin pregnancies including course of pregnancy and fetal outcome. Fetal Diagn Ther.

[4] Karacay Ö, Sepici-Dincel A, Karcaaltincaba D, Sahin D, Yalvaç S, Akyol M, et al (2010). A quantitative evaluation of total antioxidant status and oxidative stress markers in preeclampsia and gestational diabetic patients in 24-36 weeks of gestation. Diabetes Res Clin Pract.

[5] Knöfler M (2010). Critical growth factors and signalling pathways controlling human trophoblast invasion. Int J Dev Biol.

[6] Grzebyk E, Piwowar A (2013). Glycoxidative modification of albumin in medical research. Pol Merkur Lekarski.

[7] Bar-Or D, Lau E, Winkler JV (2000). A novel assay for cobalt-albumin binding and its potential as a marker for myocardial ischemia—a preliminary report1. J Emerg Med.

[8] Bahinipati J, Mohapatra PC (2016). Ischemia modified albumin as a marker of oxidative stress in normal pregnancy. J Clin Diagn Res.

[9] Üstün Y, Engin-Üstün Y, Öztürk Ö, Alanbay I, Yaman H (2011). Ischemiamodified albumin as an oxidative stress marker in preeclampsia. J Matern Fetal Neonatal Med.

[10] Tayyar AT, Kozalı S, Yetkin Yildirim G, Karakus R, Yuksel IT, Erel O, et al (2019). Role of ischemia-modified albumin in the evaluation of oxidative stress in intrahepatic cholestasis of pregnancy. J Matern Fetal Neonatal Med.

[11] Sen CK, Packer L (2000). Thiol homeostasis and supplements in physical exercise. The Am J Clin Nutr.

[12] Kemp M, Go YM, Jones DP (2008). Nonequilibrium thermodynamics of thiol/disulfide redox systems: a perspective on redox systems biology. Free Radic Biol Med.

[13] Biswas S, Chida AS, Rahman I (2006). Redox modifications of protein-thiols: emerging roles in cell signaling. Biochem Pharmacol.

[14] Circu ML, Aw TY (2010). Reactive oxygen species, cellular redox systems, and apoptosis. Free Radic Biol Med.

[15] Ozler S, Erel O, Oztas E, Ersoy AO, Ergin M, Sucak A, et al (2015). Serum thiol/disulphide homeostasis in preeclampsia. Hypertens Pregnancy.

[16] Ma S-g, Yu W-n, Jin Y, Hong B, Hu W (2012). Evaluation of serum ischemia-modified albumin levels in pregnant women with and without gestational diabetes mellitus. Gynecol Endocrinol.

[17] Cakar E, Ayvacı H, Karcaaltincaba D, Aydın G, Cilli A, Bicer C, et al (2019). Thiol-disulfide homoestasis in pregnancies with fetal growth restriction. J Matern Fetal Neonatal Med.

[18] Erel O, Neselioglu S (2014). A novel and automated assay for thiol/disulphide homeostasis. Clin Biochem.

[19] Redman C (1991). Pre-eclampsia and the placenta. Placenta.

[20] Roberts JM, Redman CW (1993). Pre-eclampsia: more than pregnancy-induced hypertension. The Lancet.

[21] Redman C, Sargent I, Staff A (2014). IFPA Senior Award Lecture: making sense of pre-eclampsia-two placental causes of preeclampsia?. Placenta.

[22] Burton G, Woods A, Jauniaux E, Kingdom J (2009). Rheological and physiological consequences of conversion of the maternal spiral arteries for uteroplacental blood flow during human pregnancy. Placenta.

[23] Burton G, Yung H-W, Cindrova-Davies T, Charnock-Jones D (2009). Placental endoplasmic reticulum stress and oxidative stress in the pathophysiology of unexplained intrauterine growth restriction and early onset preeclampsia. Placenta.

[24] Palei AC, Spradley FT, Warrington JP, George EM, Granger JP (2013). Pathophysiology of hypertension in pre‐eclampsia: a lesson in integrative physiology. Acta Physiol (Oxf).

[25] Gurusinghe S, Wallace EM, Lim R (2014). The relationship between Activin A and anti-angiogenic factors in the development of pre-eclampsia. Pregnancy Hypertens.

[26] Mandang S, Manuelpillai U, Wallace EM (2007). Oxidative stress increases placental and endothelial cell activin A secretion. J Endocrinol.

[27] Davidge ST, editor Oxidative stress and altered endothelial cell function in preeclampsia. Seminars in reproductive endocrinology; 1998: Copyright© 1998 by Thieme Medical Publishers, Inc.

[28] Villanueva I, Alva-Sánchez C, Pacheco-Rosado J (2013). The role of thyroid hormones as inductors of oxidative stress and neurodegeneration. Oxid Med Cell Longev.

[29] Cremers CM, Jakob U (2013). Oxidant sensing by reversible disulfide bond formation. J Biol Chem.

[30] Durmuş SY, Şahin NM, Ergin M, Neşelioğlu S, Aycan Z, Erel Ö (2019). How does thiol/disulfide homeostasis change in children with type 1 diabetes mellitus?. Diabetes Res Clin Pract.

[31] Karatas F, Acat M, Sahin S, Inci F, Karatas G, Neselioglu S, et al (2019). The prognostic and predictive significance of serum thiols and disulfide levels in advanced non-small cell lung cancer. The Aging Male.

[32] Unal S, Ulubas Isik D, Bas AY, Erol S, Arifoglu İ, Alisik M, et al (2019). Evaluation of dynamic thiol-disulfide homeostasis in very low-birth-weighted preterms. J Matern Fetal Neonatal Med.

[33] Üstündağ Y, Demirci H, Balık R, Erel O, Özaydın F, Kücük B, et al (2019). Thiol/disulfide homeostasis in pregnant women with obstructive sleep apnea syndrome. J Matern Fetal Neonatal Med.

[34] Jeong S-O, Pae H-O, Oh G-S, Jeong G-S, Lee B-S, Lee S, et al (2006). Hydrogen sulfide potentiates interleukin-1β-induced nitric oxide production via enhancement of extracellular signal-regulated kinase activation in rat vascular smooth muscle cells. Biochem Biophys Res Commun.

[35] Holwerda K, Bos E, Rajakumar A, Ris-Stalpers C, Van Pampus M, Timmer A, et al (2012). Hydrogen sulfide producing enzymes in pregnancy and preeclampsia. Placenta.

[36] Korkmaz V, Kurdoglu Z, Alisik M, Çetin O, Korkmaz H, Sürer H, et al (2016). Impairment of thiol disulphide homeostasis in preeclampsia. J Matern Fetal Neonatal Med.

[37] Aydin O, Ellidag HY, Eren E, Kurtulus F, Yaman A, Yilmaz N (2014). Ischemia modified albumin is an indicator of oxidative stress in multiple sclerosis. Biochem Med (Zagreb).

[38] Kilic MO, Guldogan CE, Balamir I, Tez M (2017). Ischemia-modified albumin as a predictor of the severity of acute appendicitis. Am J Emerg Med.

[39] Van Rijn BB, Franx A, Sikkema JM, van Rijn HJ, Bruinse HW, Voorbij HA (2008). Ischemia modified albumin in normal pregnancy and preeclampsia. Hypertens Pregnancy.

